# AKAP3 correlates with triple negative status and disease free survival in breast cancer

**DOI:** 10.1186/s12885-015-1668-0

**Published:** 2015-10-12

**Authors:** Rezvan Esmaeili, Keivan Majidzadeh-A, Leila Farahmand, Maryam Ghasemi, Malihe Salehi, Ali Reza Khoshdel

**Affiliations:** 1 Cancer Genetics Department, Breast Cancer Research Center, ACECR, No 146, South Gandhi Ave, Vanak Sq., Tehran, Iran; 2 Tasnim Biotechnology Research Center (TBRC), School of Medicine, AJA University of Medical Sciences, Tehran, Iran; 3 Department of epidemiology, School of Medicine, Aja University of Medical Science, Tehran, Iran

## Abstract

**Background:**

Cancer-testis antigens are among the new promising biomarkers, especially for targeted therapy. Aberrant and specific expression of these proteins has been reported in some tumor tissues. Also understanding their differential role in normal and cancer tissues may introduce them as new candidates for biomarker in cancer.

**Methods:**

AKAP3 expression was investigated in 162 tumors, normal adjacent and normal tissues of the breast with Real-Time PCR. Also the correlation between the gene expression and clinico-pathologic features of the tumors and treatment regimen was evaluated.

**Results:**

There was an association between lack of AKAP3 expression in tumor tissues and triple negative status (p=. 03). There was also a correlation between lack of this marker and tumor size (*p* = .01) and stage (*p* = .04). Lack of AKAP3 in normal adjacent tissues was associated with poor prognosis. Kaplan Meier plot demonstrated a remarkable better 5-year disease free survival in AKAP3 positive normal adjacent group.

**Conclusions:**

It was found that this relationship is originated from the difference in AKAP3 expression, not therapy distribution between two groups of patients. Thus, it may be a proper biomarker candidate for triple negative breast cancer patients. Also, testing AKAP3 in normal tissue of the patients may be used to predict the outcome of the treatment.

## Background

Among women, breast cancer is the most prevalent cancer and also one of the leading causes of cancer mortality. Biomarkers are the most useful tools for prevention and better management of the disease. Although the biomarker discovery has led to a great deal of results in many aspects of cancer, there are still many challenging issues in this area which cause biomarker discovery to be still underway. Moreover triple negative breast cancer (TNBC) as a more aggressive and poor prognosis breast cancer is still an important clinical challenge.

Cancer-testis antigens (CTA) are among the new promising biomarkers, especially for targeted therapy [1]. They are members of a group of proteins which are normally expressed in testis and to a lesser extent in ovarian germ cells [2, 3]. Since the aberrant and specific expression of these biomarkers has been reported in some tumor tissues, they may act as new candidates for targeted therapy [4]. Also understanding their differential role in normal and cancer tissues may emerge new predictive or prognostic biomarkers. Therefore the study of the expression pattern of these biomarkers and its relationship to clinical features of the patients are subjects of great interest.

A-kinase anchoring proteins (AKAP) are a group of CTA which play important roles in sperm function and are classified based on their ability of binding to c-AMP dependent protein kinase A (PKA) II. AKAP encoded proteins are localized in the fibrous sheath of sperm and may act as regulators of its motility, capacitation and acrosome reaction [5].

AKAP3 is a member of AKAP proteins that was reported to be expressed in epithelial ovary cancer. AKAP3 expression was found to be a significant predictor of both overall and progression-free survival in patients with poorly differentiated ovary tumors [6].

In this study, we aimed to determine if there is a specific expression of AKAP3 in tumor compared to normal tissue. Based on this specific expression, this marker can be a candidate to use as prognostic, predictive or even as a candidate for targeted therapy. To test this potency, the presence of AKAP3 mRNA was investigated in invasive ductal carcinoma (IDC) of breast comparing to adjacent normal and normal tissues. Also the correlation between the gene expression and clinico-pathologic features of the tumors and treatment regimen was evaluated.

## Material & methods

### Samples

A total of 162 breast tissue samples including 74 tumor, 73 normal adjacent, 15 normal breast tissues were taken from the Breast Cancer Research Center Biobank (BCRC-BB) [7]. Two breast cancer cell lines (MCF7 and T47D) (taken from Avicenna infertility clinic (AIC)) were included in the study. To check the probability of AKAP3 expression in breast tumor cells, its expression was checked first in these two cell line then it was assayed in tumor, normal and normal adjacent tissues using Normal testis tissue as a positive control. According to the protocols followed by BCRC-BB, after excisional biopsy or surgery the content of cancer cells in each sample was pathologically checked and immediately sample tissues were snap-frozen in liquid nitrogen and stored at −70 °C. BCRC-BB is obliged to ethical guidelines and recommendations for biobanks on the storage and use of human biological samples. Also, all patients provided written informed consent before entering the biobank. This study was approved by the Ethics Committee of the BCRC before conducting the project. The Clinical and histopathological features of patients along with treatment regimen were gathered. Patient’s status was defined based on the occurrence of any event like recurrence, metastasis or death due to cancer in their follow-up history. Patients who had these events are categorized as poor prognosis group. Patients were categorized in treatment subgroups according their treatment status as follows: CMF (cyclophosphamide, methotrexate and 5-fluorouracil) regimen and anthracycline and/or taxane-containing regimen with or without endocrine therapy and finally endocrine therapy only.

### Real-Time PCR

RNA extraction and cDNA synthesis were done as previously explained [8]. Primers for AKAP3 and Actin Beta (ACTB) which was designed by Gene Runner v.3.05 and confirmed with primer express 3.0.are as follows: AKAP3 F: CAGGACTGGAAAATGGACACCT, AKAP3 R: TTTGTGTGGGTCTCCTGAGTTG. ACTB F: CAGCAGATGTGGATCAGCAAG, ACTB R: GCATTTGCGGTGGACGAT

To check the presence of AKAP3 mRNA, Real Time PCR was carried out using SYBR Green PCR Master Mix (PrimerDesign Ltd, UK). Primer concentration was 0.5 μM for both genes. ACTB was detected to confirm cDNA quality. Fluorescent detection was performed using Applied Biosystems 7500 System. To ensure the formation of specific amplicon, first the size of the PCR product was checked on agarose electrophoresis and was sequenced using Big Dye terminator DNA sequencing (Applied Biosystems, Foster City, CA) (data not shown), then each reaction was followed by melting curve analysis involving heating of the PCR product from 60 to 95 °C. The curve in the specimens was compared with curve of positive control to recognize specific amplicon from primer dimer formation. The melt point of 83.4 °C was defined for AKAP3 amplicon. Reactions with this melting point were considered positive for AKAP3 expression. Any amplicon with other melt point was regarded as nonspecific amplification. Data were analyzed using SDS software, vers.2.0 (Applied Biosystems).

### Data analysis

Patients were categorized based on their type of tumor, according to ER, PR and Her2/neu status. Moreover AKAP3 expression was analyzed in each type of tumor, including triple negative group and the remaining types including ER/PR + Her2/neu -, ER/PR- Her2/neu + and ER/PR/ Her2/neu +. Different combinations of these groups were used for data analysis.

All statistical analyses were performed with the SPSS statistical software (vers.18). The relationship between the presence of AKAP3 expression and clinico-pathological data and treatment regimen were analyzed using nonparametric tests. Patient disease free survival was assessed by Kaplan-Meier analysis using log-rank tests. The P value of <0.05 was considered as significant results. All the plots were performed using Graph Pad Prism 3.02 (GraphPad Software, San Diego, Calif).

## Results

### Study population

Clinico-pathological characteristics of the study population are presented in Table 1. The median age of the samples was 48 (range: 29–87 years). Median duration of follow up was 35 (1–65 months). The percentage of patients with stage 1 to 4 is 10, 48, 30 and 12 correspondingly. 31 % of patients had an event like recurrence, metastasis or death due to cancer in their follow-up history while 69 % didn’t show any event and 4 patients had no available event data. ER, PR and Her2/neu positivity were 44, 35 and 31% respectively. The most frequent treatment subgroup was anthracycline and/or taxane-containing regimen with endocrine which was 44% (data not shown).

**Table 1:** Patient’s characteristics and AKAP3 status. Stage grouping are based on American Joint Committee on Cancer (AJCC), Estrogen receptor (ER), progesterone receptor (PR), Her2/neu are based on IHC results. Cut point of positivity is based on American Society of Clinical Oncology (ASCO) guideline for IHC

Number of patients	74	
Age (median/range) — year	48 (29–87)	Valid percent
Follow-up (median/range)—month	1 8 (1–38)	
ER status (2 missing, n = 74)		
Negative	40	55
Positive	32	45
PR (2 missing, n = 74)		
Negative	47	65
Positive	25	35
Her2/neu status (2 missing, n = 74)		
Negative	50	69
Positive	22	31
Grade (7 missing, n = 74)		
G1	6	9
G2	34	51
G3	27	40
Patient status (3 missing, n = 74)		
healthy survival	50	70
with events	21	30
Stage (5 missing, n = 74)		
I	7	10
II	33	48
III	21	30
IV	8	12
Tumor Size (6 missing, n = 74)		
<=2	24	35
>2	44	65
AKAP3 Tumor (n = 74)		
Negative	47	64
Positive	27	36
AKAP3 Normal adjacent (n = 73)		
Negative	40	56
Positive	32	44
Akap3 Normal Breast (n = 15)		
Negative	8	54
Positive	7	46

### Expression of AKAP3 mRNA

Two breast cancer cell lines expressed AKAP3 mRNA. AKAP3 expression was seen in normal testis tissue which serves as a positive control, 36.5 % of tumors, 43.2 % of normal adjacent and 46.6 % of normal breast tissues, while Actin Beta was expressed in all the samples.

The McNemar’s test revealed no difference in distribution of AKAP3 expression between tumor and normal adjacent tissues. The relationship between AKAP3 expression and ER, PR and Her2/neu status was analyzed in tumor and normal adjacent tissues separately. There was a significant association between lack of AKAP3 expression in tumor tissues and triple negative status (p=. 03) and there was no correlation with the remaining types including ER/PR + Her2/neu -, ER/PR- Her2/neu + and ER/PR/ Her2/neu +. AKAP3 negativity is a real absence of this gene and it is not based on specific cut point. To check if this difference originates from AKAP3 expression or from treatment regimen, the association between AKAP3 expression in tumor tissues and treatment regimen was investigated and no association was seen.

**Figure 1:**
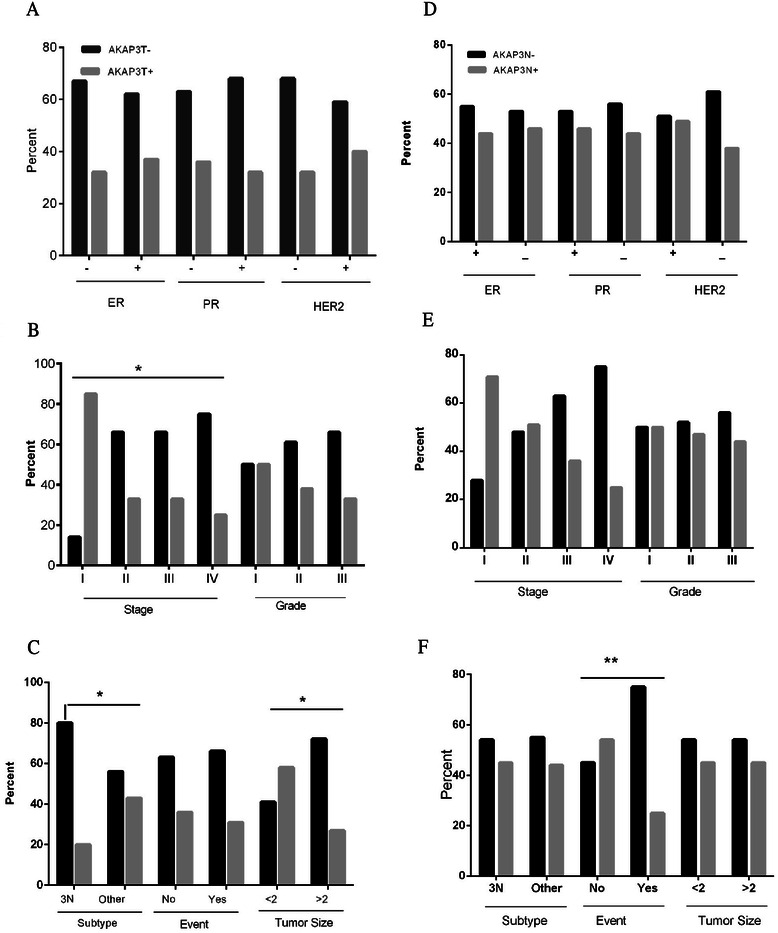
AKAP3 expression in tumor and normal adjacent tissue of the breast. **a**-**c** Represent AKAP3 expression in tumor (AKAP3T) and clinico-pathological features. Significant results were seen in stage, subtype and tumor size. **d-f** Show AKAP3 expression in normal adjacent tissue (AKAP3N) and clinico-pathological features. Significant result was seen in the event which represents prognosis

**Figure 2:**
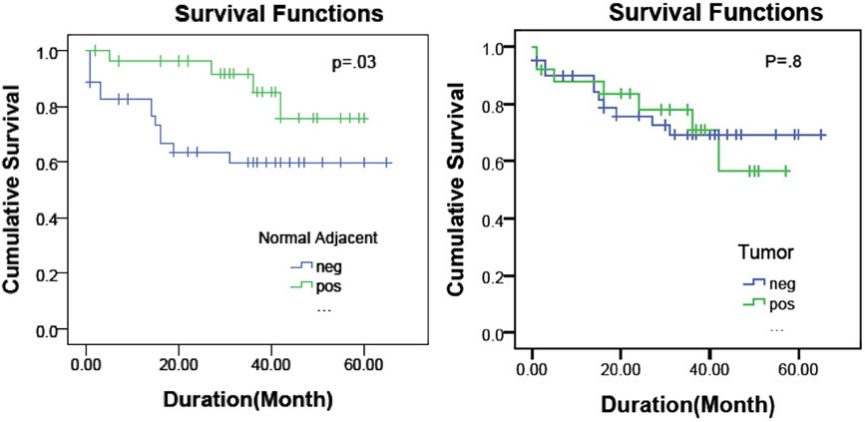
Kaplan Meier plot. Kaplan Meier plot of patient survival stratified by AKAP3 expression in tumor (right) and normal adjacent (left) tissues. Vertical tick marks represent censored patients. Censoring means the total survival time for that patient cannot be accurately determined. Most of the time occurs when participants either drop out of the study or refuse to participate in the study

There was a significant association between lack of AKAP3 in normal adjacent tissue and poor prognosis (*p* = .003). It means that patients with AKAP3 expression in normal adjacent tumor (28/63 of the patients) were associated with only 4/28 events compared to an event rate of 13/35 in the AKAP3 negative group. Kaplan Meier plot demonstrated a remarkable better survival in AKAP3 positive normal adjacent group, that was statistically significant in log-rank analysis (*P* = .03) (Fig. 1). The follow-up for both groups were comparable (*P* = .2). There was not such a relationship with the AKAP3 expression in tumor tissues (*P* = .8). To find if this significance result originated from triple negative status as a poor prognosis subtype or it is from lack of AKAP3 expression in normal adjacent, we analyzed the relationship of AKAP3 in normal adjacent tissues and prognosis in the triple negative group compared to other types. This association was not correlated with the triple negative group. In other words, the relationship between lack of AKAP3 expression in normal adjacent and poorer outcome is significantly detectable in the other subtypes of breast cancer than triple negative breast cancer (*p* = .01). The analysis between AKAP3 expression in normal adjacent tissues and treatment regimen didn't show any significant results (Fig. 2). Chi-square analysis showed a significant relationship between the expression of AKAP3 in tumor tissues and tumor size (p=. 01) and stage (p=. 04). Based on these analyses, AKAP3 expression in tumor tissues was diminished in tumor size above 2 cm. Also the proportion of patients in stage one who expressed AKAP3 in their tumor tissues was more than other stages. Same analysis with grade, number of positive lymph nodes and the age of diagnosis of the patients did not show any significant results (Table 2).

**Table 2:** AKAP3 expression and clinico-pathologic data. Statistical association between clinico-pathologic data and AKAP3 expression in tumor and normal adjacent tissue

Variable	AKAP3 expression		p value	AKAP3 expression		p value
	In tumor			In normal		
	Negative no (%)	Positive no (%)		Negative no (%)	Positive no (%)	
ER						
Negative	27 (67.5)	13 (32.5)	0.4	21 (55.3)	17 (44.7)	0.5
Positive	20 (62.5)	12 (37.5)		17 (53.1)	15 (46.9)	
PR						
Negative	30 (63.8)	17 (36.2)	0.4	24 (53.3)	21 (46.7)	0.5
Positive	17 (68)	8 (32)		14 (56)	11 (44)	
Her2						
Negative	34 (68)	16 (32)	0.3	25 (51)	24 (49)	0.2
Positive	13 (59.1)	9 (40.9)		13 (61.9)	8 (38.1)	
Tumor Grade						
1	3 (50)	3 (50)	0.7	3 (50)	3 (50)	0.9
2	21 (61.8)	13 (38.2)		18 (52.9)	16 (47.1)	
3	18 (66.7)	9 (33.3)		14 (56)	11 (44)	
Lymph Node						
No	15 (68.2)	7 (31.8)	0.7	10 (45.5)	12 (54.5)	0.3
1-3	13 (56.5)	10 (43.5)		11 (47.8)	12 (52.2)	
4-9	8 (57.1)	6 (42.9)		9 (75)	3 (25)	
>9	7 (70)	3 (30)		6 (60)	4 (40)	
Tumor size						
≤2cm	10 (41.7)	14 (58.3)	0.01	13 (54.2)	11 (45.8)	0.5
>2 cm	32 (72.7)	12 (27.3)		23 (54.8)	19 (45.2)	
Stage						
I	1 (14.3)	6 (85.7)	0.04	2 (28.6)	5 (71.4)	0.2
II	22 (66.7)	11 (33.3)		16 (48.5)	17 (51.5)	
III	14 (66.7)	7 (33.3)		12 (63.2)	7 (36.8)	
IV	6 (75)	2 (25)		6 (75)	2 (25)	
Subtype						
Triple Negative	20 (80)	5 (20)	0.03	13 (54.2)	11 (45.8)	0.5
Other types	27 (56.3)	21 (43.8)		26 (55.3)	21 (44.7)	
Patient status						
No event	31 (63.3)	18 (36.7)	0.5	19 (41.3)	27 (58.7)	0.003
With event	14 (66.7)	7 (33.3)		16 (80)	4 (20)	

## Discussion

Looking for genes that are specifically expressed in cancer tissues and have a limited expression in normal tissues is one of the most important targets of researchers in the field of cancer treatment. Among these targets, CTAs could be a promising option and there are a lot of researches in this field [1, 2, 7–9]. In this study expression of AKAP3 were investigated in tumor, normal adjacent and normal tissue of the breast in association with clinical features of the patients.

AKAP3 is classified as one of the members of scaffold proteins (AKAPs). They are a family of about 50 scaffolding proteins which anchor protein kinase A (PKA) II and other proteins including protein kinases, protein phosphatases and phosphodiesterases. So they can specify intracellular locations and restrict the corresponding enzymatic activities [10, 11]. As a result of multi-protein complexes which are formed by these anchoring proteins, cAMP signaling is integrated with other pathways [5]. Such a compartmentalization represents the critical roles of AKAPs in cellular activities. Accordingly subtle changes in the AKAPs related pathways or their components is an explanation of the development of many diseases, especially cancer [12].

Triple negative breast cancer is still poorly characterized at the molecular level and Lack of prognostic markers and selective targets of therapy make it the most aggressive type of breast cancer which deserves more investigation [13, 14]. In the current study AKAP3 expression is deficient significantly in triple negative tumors. CAMP-dependent protein kinase is usually present in tissues as a mixture of type I and type II isozyme and changing their ratio involved in the oncogenic process. The PKAII is mostly present in normal tissues, whereas its isoform PKAI which is not related to AKAPs is expressed in malignant tissues [15]. Since AKAP3 is a scaffold for binding of PKAII, lack of AKAP3 expression which is seen in this study may be due to the absence or decreased level of PKAII in malignant tissues. Although this could be the reason of absence of AKAP3 expression in malignant tissue, we still don’t know whether the hormone independence status in triple negative tissues affects this status or not. If this protein have eligibility to use as prognostic factor in triple negative patient require further investigation. Based on this study, AKAP3 expression decreased significantly while tumors increased in size and stage. This may be due to the association of PKAII with inhibition of mitogenic signaling and cell growth [16]. Moreover, since PKA signaling with different AKAPs may direct cell toward proliferation or death, AKAP3 may act as an inhibitor of proliferation signals [17]. But, further study need to decide about this suggestion because down regulated genes are not always involved in inhibition of proliferation.

Based on the results, the patients with lack of AKAP3 in their normal adjacent tissues represent the poorer outcome. The analysis between expression of this gene and different treatment subtypes revealed no significant results which may show that the significant differences in patient survival, found in this study, would be originated from the difference in AKAP3 expression not therapy distribution between two groups of patients. In the previous study by Sharma s et al., AKAP3 expression correlated with poor prognosis in epithelial ovarian cancer [6], but its expression was not explored in normal adjacent tissue. Alteration in gene expression of histologically normal adjacent tissue is a matter of debate. A number of studies suggested that morphologically normal tissue adjacent to carcinomas has not undergone significant gene expression changes when compared to normal tissue [18]. In contrast, some evidence suggests that these tissues represent modifications in molecular level resembling tumor tissues [19]. These alterations can reveal the earliest changes leading to tumorigenesis or may due to the effect of the tumor. In two studies in prostate cancer, microarray comparison of tumor, normal adjacent and normal tissue revealed up- regulation of transcription factors, signal transducers and growth regulatory genes in tumor and normal adjacent but normal tissues [20, 21]. In 2006 Schneiders et al., suggested that the genetic profile of the normal adjacent tissue may be associated with treatment failure in rectal cancer, suggesting the importance of the tumor microenvironment in the development of recurrence of rectal tumors [22]. Since recurrence is a significant clinical problem for patients with cancer, having the possibility of genetic assessment in normal material of patients in predicting the recurrence is of great interest in medical research. More research on AKAP3 on easily accessible tissues of the patients could help to decide whether it is possible to use normal material of the patients to predict the outcome of the treatment.

## Conclusion

In conclusion, between the variety of biomarkers candidates, CTAs are promising genes among which AKAPs draw much attention. Based on the results, AKAP3 may be a proper candidate for further investigation as a biomarker for triple negative status. AKAP3 may act as inhibitor of proliferation since it was not expressed in higher stage and tumor size. Moreover, our findings warrant complementary experiments, especially in protein level to define the value of AKAP3 testing in normal tissue of patients as a marker of response to treatment.

## Abbreviations

AKAP: A-kinase anchoring proteins
TNBC: Triple negative breast cancer
CTA: Cancer-testis antigens
PKA: Protein kinase A
IDC: Invasive ductal carcinoma
BCRC-BB: Breast Cancer Research Center Biobank
CMF: Cyclophosphamide, methotrexate and 5-fluorouracil
ER: Estrogen receptor
PR: Progesterone receptor
IHC: Immunohistochemistry
ASCO: American Society of Clinical Oncology
